# Supplementary material for: Pyruvate Dehydrogenase Kinase 1 inhibition mediated oxidative phosphorylation enhancement in cartilage promotes osteoarthritis progression
Source: BMC Musculoskelet Disord. 2023 Jul 20;24:597. doi: 10.1186/s12891-023-06585-6 (PMC10357736; doi:10.1186/s12891-023-06585-6)
Supplement: Supplementary file 1 — Additional file 1. [file 12891_2023_6585_MOESM1_ESM.zip › MF.Gsea.1653627316242/GOMF_NEUROPEPTIDE_BINDING.html]

Details for gene set GOMF\_NEUROPEPTIDE\_BINDING[GSEA]

|  || Dataset | 55457\_collapsed\_to\_symbols.cls.cls #OA\_versus\_normal.cls.cls #OA\_versus\_normal\_repos |
| Phenotype | cls.cls#OA\_versus\_normal\_repos |
| Upregulated in class | normal |
| GeneSet | GOMF\_NEUROPEPTIDE\_BINDING |
| Enrichment Score (ES) | -0.54361826 |
| Normalized Enrichment Score (NES) | -1.7010331 |
| Nominal p-value | 0.011705685 |
| FDR q-value | 0.10612699 |
| FWER p-Value | 0.899 |
Table: GSEA Results Summary

  

Fig 1: Enrichment plot: GOMF\_NEUROPEPTIDE\_BINDING      
 Profile of the Running ES Score & Positions of GeneSet Members on the Rank Ordered List

  

| SYMBOL | TITLE | RANK IN GENE LIST | RANK METRIC SCORE | RUNNING ES | CORE ENRICHMENT || 1 | CMKLR2 | chemerin chemokine-like receptor 2 [Source:HGNC Symbol;Acc:HGNC:4463] | 1439 | 0.437 | -0.0564 | No |
| 2 | SSTR4 | somatostatin receptor 4 [Source:HGNC Symbol;Acc:HGNC:11333] | 1597 | 0.414 | -0.0161 | No |
| 3 | OPRL1 | opioid related nociceptin receptor 1 [Source:HGNC Symbol;Acc:HGNC:8155] | 3166 | 0.234 | -0.1082 | No |
| 4 | OPRD1 | opioid receptor delta 1 [Source:HGNC Symbol;Acc:HGNC:8153] | 3663 | 0.189 | -0.1228 | No |
| 5 | GPR37 | G protein-coupled receptor 37 [Source:HGNC Symbol;Acc:HGNC:4494] | 5474 | 0.035 | -0.2589 | No |
| 6 | MC3R | melanocortin 3 receptor [Source:HGNC Symbol;Acc:HGNC:6931] | 5910 | 0.002 | -0.2925 | No |
| 7 | MC4R | melanocortin 4 receptor [Source:HGNC Symbol;Acc:HGNC:6932] | 5918 | 0.001 | -0.2929 | No |
| 8 | SORL1 | sortilin related receptor 1 [Source:HGNC Symbol;Acc:HGNC:11185] | 6143 | -0.016 | -0.3083 | No |
| 9 | MCHR1 | melanin concentrating hormone receptor 1 [Source:HGNC Symbol;Acc:HGNC:4479] | 6242 | -0.024 | -0.3130 | No |
| 10 | OPRM1 | opioid receptor mu 1 [Source:HGNC Symbol;Acc:HGNC:8156] | 6351 | -0.032 | -0.3173 | No |
| 11 | GRPR | gastrin releasing peptide receptor [Source:HGNC Symbol;Acc:HGNC:4609] | 7361 | -0.107 | -0.3821 | No |
| 12 | OPRK1 | opioid receptor kappa 1 [Source:HGNC Symbol;Acc:HGNC:8154] | 9046 | -0.235 | -0.4831 | Yes |
| 13 | SSTR5 | somatostatin receptor 5 [Source:HGNC Symbol;Acc:HGNC:11334] | 9272 | -0.253 | -0.4685 | Yes |
| 14 | NPBWR2 | neuropeptides B and W receptor 2 [Source:HGNC Symbol;Acc:HGNC:4530] | 9622 | -0.285 | -0.4595 | Yes |
| 15 | SSTR1 | somatostatin receptor 1 [Source:HGNC Symbol;Acc:HGNC:11330] | 9803 | -0.302 | -0.4353 | Yes |
| 16 | PTGDR2 | prostaglandin D2 receptor 2 [Source:HGNC Symbol;Acc:HGNC:4502] | 10753 | -0.399 | -0.4584 | Yes |
| 17 | GALR1 | galanin receptor 1 [Source:HGNC Symbol;Acc:HGNC:4132] | 11851 | -0.560 | -0.4727 | Yes |
| 18 | GALR2 | galanin receptor 2 [Source:HGNC Symbol;Acc:HGNC:4133] | 12072 | -0.612 | -0.4122 | Yes |
| 19 | SSTR2 | somatostatin receptor 2 [Source:HGNC Symbol;Acc:HGNC:11331] | 12181 | -0.639 | -0.3396 | Yes |
| 20 | SSTR3 | somatostatin receptor 3 [Source:HGNC Symbol;Acc:HGNC:11332] | 12779 | -0.936 | -0.2675 | Yes |
| 21 | ADCYAP1R1 | ADCYAP receptor type I [Source:HGNC Symbol;Acc:HGNC:242] | 12790 | -0.952 | -0.1477 | Yes |
| 22 | NMUR1 | neuromedin U receptor 1 [Source:HGNC Symbol;Acc:HGNC:4518] | 12887 | -1.233 | 0.0010 | Yes |
Table: GSEA details [plain text format]

  

Fig 2: GOMF\_NEUROPEPTIDE\_BINDING      
 Blue-Pink O' Gram in the Space of the Analyzed GeneSet

  

Fig 3: GOMF\_NEUROPEPTIDE\_BINDING: Random ES distribution      
 Gene set null distribution of ES for **GOMF\_NEUROPEPTIDE\_BINDING**
